# Effect of Warm-Mix Additive USP on the Performance of Rubberized Asphalt and Fiber-Reinforced Rubberized Asphalt RAP Interlayer

**DOI:** 10.3390/polym17192616

**Published:** 2025-09-27

**Authors:** Jianhang Han, Bin Ding, Yong Hua, Wenbo Liu, Jun Li

**Affiliations:** 1 China First Highway Engineering Co., Ltd., Beijing 101100, China; 18132825532@163.com (J.H.); 17631011677@163.com (B.D.); 18856612396@163.com (Y.H.); 2 School of Materials Science and Engineering, Chang’an University, Xi’an 710061, China; 2023131077@chd.edu.cn

**Keywords:** rubberized asphalt, interlayer, RAP, dynamic shear rheometer (DSR), crack resistance

## Abstract

To address the dual challenges of cryogenic performance degradation and excessive VOC emissions in rubberized asphalt, this study proposes a synergistic optimization strategy using a polymer-based warm-mix additive (USP). The effects of USP on the rheological behavior, VOC emission characteristics, and mechanical performance of polymer-modified asphalt and fiber-reinforced RAP interlayers were systematically investigated. The results indicate that 5% USP optimally improves low-temperature flexibility (141.1% increase in ductility, 28.48% reduction in creep stiffness) while maintaining adequate high-temperature stability, and simultaneously achieves an 82.01% reduction in total VOC emissions at 150 °C. Microscopic analysis and DIC tests confirm that USP enhances polymer–asphalt–aggregate interactions, leading to improved adhesion, reduced water permeability, and extended fatigue life. This work provides a fundamental understanding of polymer–binder–aggregate synergy and offers a practical pathway toward greener, high-performance recycled asphalt pavement technologies.

## 1. Introduction

With the continuous growth of traffic loading and increasingly extreme climate conditions, premature pavement failures have become a pressing issue, which has driven extensive research into asphalt modification technologies. Commonly used asphalt modifiers include polymers, fibers, nanomaterials, and industrial by-products, each with distinct advantages and limitations [1]. Rubber asphalt has emerged as a highly promising polymeric binder in road engineering due to its unique entropy-driven elastic recovery capabilities and superior fatigue resistance, finding extensive application in fiber-reinforced crushed stone layers [2,3]. By incorporating crumb rubber particles (a vulcanized elastomer) from waste tires, rubber asphalt not only significantly enhances pavement crack resistance, noise reduction, and durability through enhanced polymer network formation but also provides an effective pathway for solid waste recycling [4]. However, the high-temperature construction requirements of this polymer-modified binder pose two critical technical challenges: First, in cold regions, rapid heat dissipation during construction leads to abrupt chain mobility reduction and viscosity increases, resulting in uneven aggregate coating and reduced interfacial bonding strength. Second, high-temperature mixing processes release substantial volatile organic compounds (VOCs), including carcinogens such as benzene homologs and polycyclic aromatic hydrocarbons (PAHs), posing severe threats to ecological environments and construction worker safety [5,6]. This contradiction between “polymer chain immobilization and performance degradation under low-temperature construction” and “heightened polymer degradation and environmental risks under high-temperature conditions” severely limits the broader application of rubber asphalt in extreme climates.

Against this backdrop, RAP fiber interlayer technology has garnered significant attention for its environmental and economic benefits [7,8]. By replacing natural aggregates with RAP and incorporating fiber reinforcement [9,10], this technology simultaneously achieves recycled pavement material utilization and enhanced structural performance. Our previous studies confirmed that when basalt fiber content reaches 80 g/m^2^ and RAP replacement ratio attains 30%, the interlayer exhibits marked improvements in bonding strength, flexural strength, and fatigue resistance [11]. However, oxidized and aged polymeric components (asphalt) in RAP materials induce binder property fluctuations due to polymer degradation, particularly under low temperatures, where brittle debonding at the fiber-polymer interface triggers reflective crack propagation [12,13]. Furthermore, existing fiber reinforcement systems still rely on high-temperature construction processes, failing to fundamentally resolve VOC emissions and hindering large-scale applications [14,15].

In recent years, polymer-based USP admixture technology has offered novel insights into reconciling green construction and high-performance asphalt pavement [16,17,18]. USP reduces construction temperatures by modifying the polymer chain interactions and thus lowering high-temperature viscosity, thereby decreasing energy consumption and hazardous emissions while improving workability [19]. However, current research predominantly focuses on USP’s effects on conventional asphalt [20], leaving its polymeric interaction mechanisms within the complex rubber asphalt systems poorly understood. Key scientific gaps persist: First, the threshold effects of USP admixture content on rubber asphalt’s polymer network rheology remain unquantified, complicating optimal dosage determination in practice [21]. Second, the emission characteristics of VOCs during USP modification and their coupling relationships with polymeric material performance are undefined, obstructing synergistic optimization of environmental and mechanical performance. These knowledge gaps force engineers to choose between “sacrificing performance for emission reduction” or “compromising sustainability for performance,” underscoring the urgent need to elucidate USP’s polymeric modification mechanisms and eco-mechanical synergies in rubber asphalt systems.

**Figure 1:**
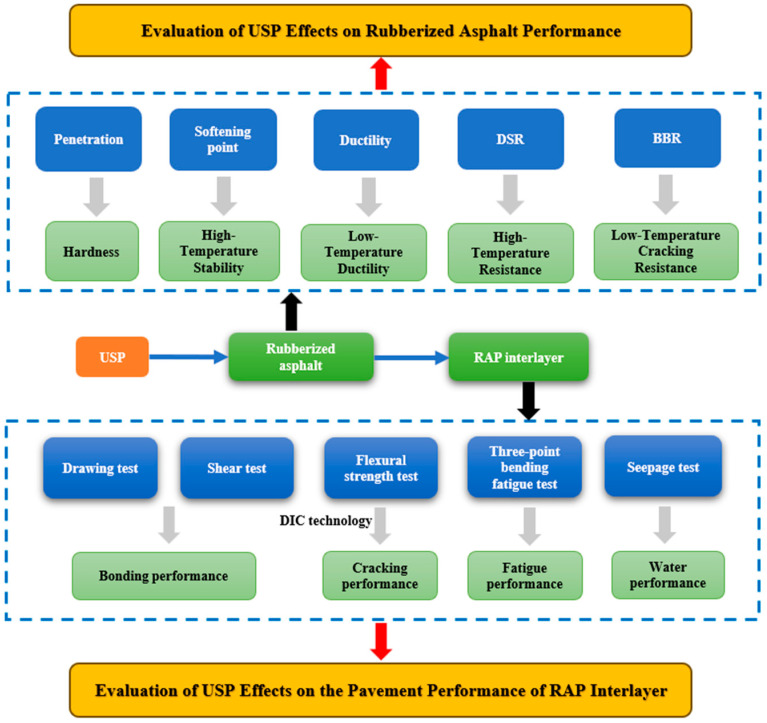
Research process of the paper.

To address these challenges, this study proposes a USP admixture-enabled collaborative optimization strategy based on pre-optimized RAP composite interlayer systems, aiming to unravel USP’s regulatory mechanisms on polymeric material performance and environmental impacts. Through conventional binder tests (penetration, softening point, ductility) combined with high-/low-temperature rheological analyses using dynamic shear rheometry (DSR) and bending beam rheometry (BBR), we systematically investigate USP’s effects on the polymer-modified binder’s viscoelastic properties, establishing quantitative models between admixture content and performance to clarify threshold effects on low-temperature cracking resistance and high-temperature rutting resistance. Furthermore, gas chromatography-mass spectrometry (GC-MS) quantifies USP’s efficacy in reducing hazardous VOCs derived from polymer degradation during recycling, unveiling its environmental modification mechanisms. Additionally, modified pull-out tests, direct shear tests, permeability tests, three-point bending tests, and fatigue tests comprehensively evaluate USP’s enhancements in interfacial bonding strength, moisture resistance, crack resistance, and durability. Digital image correlation (DIC) dynamically monitors crack propagation under loading. Detailed testing protocols are illustrated in Figure 1 and designed to correlate performance with the underlying polymeric structure.

The novelty of this study lies in three main aspects: (i) it systematically investigates, for the first time, the threshold effects of USP dosage in a rubber asphalt–RAP fiber composite system and clarifies its synergistic regulation mechanisms on high- and low-temperature rheological properties; (ii) it quantitatively reveals the suppressive effects of USP on VOC emissions during recycling and elucidates their coupling relationship with material performance; and (iii) it proposes a collaborative optimization strategy that simultaneously achieves green construction and enhanced mechanical properties, providing a theoretical and practical basis for the large-scale sustainable application of rubberized asphalt in cold regions.

## 2. Materials and Experimental Methods

### 2.1. Materials

#### 2.1.1. Binder

This study employs rubber asphalt as the binder material, with its key performance parameters summarized in Table 1. In this study, 90# base asphalt (Sinopec Xi’an Company, Xi’an, China) was used as the matrix for preparing rubberized asphalt.

#### 2.1.2. Aggregate

The basalt aggregate (Shaanxi Lantian Stone Co., Xi’an, China) used in this study has a particle size range of 5–10 mm and a moisture content of 0.42%. The cumulative sieve residue of the aggregate is illustrated in Figure 1, with key performance parameters listed in Table 2.

#### 2.1.3. RAP

**Figure 2:**
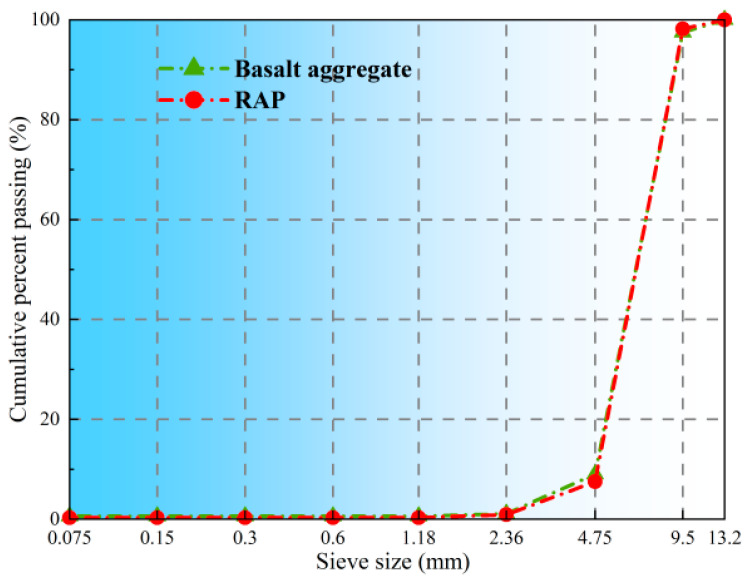
Gradation of basalt aggregate and RAP.

The RAP material (Xi’an Road Material Recycling Plant, Xi’an, China) exhibits a moisture content of 0.62% and an asphalt content of 4.9%. The properties of the aged asphalt in RAP are detailed in Table 1. Figure 2 presents the gradation curve of the RAP material.

#### 2.1.4. Basalt Fiber

The physical characteristics of the basalt fiber (Shaanxi Chang’an Advanced Fiber Co., Xi’an, China) are summarized in Table 3.

#### 2.1.5. USP Warm-Mix Modifier

The USP warm-mix modifier (Shaanxi Road Construction Materials Co., Xi’an, China) is a novel asphalt additive primarily composed of waste rubber particles, recycled plastics, and organic additives. Its key feature lies in effectively reducing the mixing and compaction temperatures of asphalt mixtures. The technical specifications of this modifier are provided in Table 4.

### 2.2. Material Preparation

#### 2.2.1. Laboratory Preparation of USP Warm-Mix Modified Rubberized Asphalt

The laboratory preparation of USP-modified rubberized asphalt comprised two sequential phases: rubberized asphalt matrix synthesis and low-temperature modifier blending. Initially, the base asphalt was heated to 175–195 °C under constant temperature control. Crumb rubber particles (finer than 20 mesh) were then incorporated, followed by continuous high-speed shearing at 3000–4000 rpm for 90 min. Real-time temperature monitoring via embedded sensors ensured strict thermal stability within the 175–195 °C range to prevent sulfur devulcanization or rubber depolymerization. Upon completing the matrix synthesis, the system temperature was systematically lowered to 150–160 °C for the modifier blending stage. USP modifier was gradually introduced at predefined dosages (0%, 4%, 5%, 6%, 7%) under low-speed mechanical stirring (200 rpm) for 20 min, achieving uniform dispersion while mitigating material aging or volatile emissions. The mix design of USP warm-mix modified rubberized asphalt is shown in Table 5. (The USP content was 0 wt.%, 4 wt.%, 5 wt.%, 6 wt.%, 7 wt.% per 100 wt.% of the mixture of base asphalt and crumb rubber).

#### 2.2.2. Fabrication of USP-Modified RAP Interlayer Specimens

The fiber-reinforced RAP interlayer was constructed as a four-layer composite system, sequentially comprising a bottom USP-modified rubberized asphalt binder layer, a basalt fiber reinforcement layer, a secondary USP-modified asphalt binder layer, and a surface aggregate scattering layer. RAP partially substituted conventional crushed stone aggregates in the interlayer structure.

For shear and pull-out testing, standard Marshall specimens (Φ152.4 mm × 40 mm) simulating actual pavement layers were prepared according to Standard Test Methods for Asphalt and Asphalt Mixtures in Highway Engineering. A 35 mm-thick asphalt mixture base layer was first compacted, followed by the USP-modified RAP interlayer construction and a 35 mm-thick surface layer overlay.

Permeability, flexural strength, and fatigue test specimens were fabricated as 300 mm × 300 mm × 80 mm composite slabs. After laying a 35 mm-thick asphalt mixture base, the USP-modified RAP interlayer was integrated and sealed with an additional 35 mm-thick asphalt layer. These slabs were subsequently cut into 250 mm × 80 mm × 80 mm standard beams, each featuring a 15 mm-deep artificial notch at the midspan bottom surface to predefine crack initiation points.

### 2.3. Experimental Methods

#### 2.3.1. Three Fundamental Binder Tests

Penetration, ductility, and softening point serve as critical indicators for evaluating asphalt performance. In accordance with the Standard Test Methods for Asphalt and Asphalt Mixtures in Highway Engineering (JTG E20-2011), this study conducted these tests on both rubberized asphalt and USP-modified rubberized asphalt to investigate the influence of the USP warm-mix modifier on binder properties. All test results were evaluated against the Technical Specifications for Rubber Asphalt Pavements (CJJ/T 273-2019) to ensure compliance with industry standards [25].

#### 2.3.2. Bending Beam Rheometer (BBR) Test

The low-temperature performance of rubberized asphalt and its USP-modified variants was assessed using a bending beam rheometer (BBR) following JTG E20-2011. Tests were performed under three temperature conditions (−12 °C, −18 °C, and −24 °C), with three parallel replicates per sample. Creep stiffness (S, MPa) and creep rate (m-value) were measured to systematically evaluate the improvement in low-temperature crack resistance achieved through USP modification.

#### 2.3.3. Dynamic Shear Rheometer (DSR) Test

Rheological properties of the asphalt binders were characterized using a dynamic shear rheometer (DSR) in compliance with JTG E20-2011. Testing spanned a temperature range of 45 °C to 90 °C at 5 °C intervals, with three replicates per temperature point. Key parameters, including complex shear modulus (G), phase angle (δ), and rutting factor (G/sinδ), were determined to quantify the USP modifier’s effectiveness in enhancing high-temperature performance and resistance to permanent deformation.

#### 2.3.4. VOCs Emission Test

**Figure 3:**
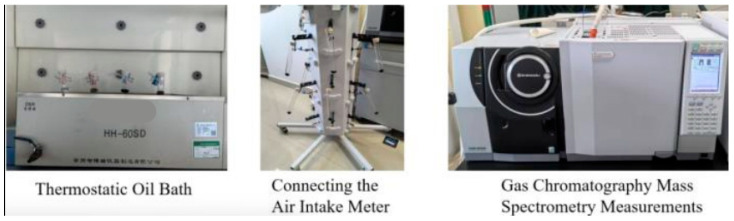
Test procedure of VOCs Emission.

This study analyzed the impact of USP admixture content on volatile organic compound (VOC) emissions and construction temperature reduction. The experimental design included two control groups and four experimental groups: Control Group 1 consisted of unmodified rubberized asphalt tested at 170 °C to simulate conventional construction conditions, while Control Group 2 evaluated baseline VOC emissions at 150 °C. Experimental groups incorporated 4%, 5%, 6%, and 7% USP admixture and were tested at 150 °C to mimic low-temperature construction. Three parallel samples per group ensured data reliability. The procedure involved placing 1 g of rubberized asphalt in a 100 mL branched test tube equipped with an air valve, sealing the system, and heating it in an oil bath at either 150 °C or 170 °C for 30 min. Emitted VOCs were collected and analyzed via GC-MS under specified conditions: an ion source temperature of 230 °C, full-scan electron ionization mode (*m*/*z* 22.5–400), and a three-stage temperature program (30 °C held for 3.2 min, ramped at 11 °C/min to 200 °C with a 3-min hold, then further ramped to 280 °C with a 10-min hold). The experimental setup is illustrated in Figure 3.

#### 2.3.5. Pull-Out Test

To evaluate the interfacial bonding performance of the RAP interlayer between the surface and base layers, pull-out tests were conducted. Marshall specimens prepared as per Section 2.2 were drilled with 96 mm-diameter, 50 mm-height holes penetrating the interlayer without fully perforating the specimen, ensuring failure occurred at the interlayer. Specimens were bonded to the testing apparatus and cured at 25 °C for 24 h. Tensile load was applied at 50 mm/min using an HTHY-0678 adhesion tester(Guangdong Huatong Testing Equipment Co., Guangzhou, China), with peak load at failure recorded to calculate tensile strength. Results were averaged across three replicates.

#### 2.3.6. Shear Strength Test

Shear resistance of the interlayer was assessed using the HTHY-0678 adhesion tester. Specimens prepared following Section 2.2 were subjected to vertical stress at 50 mm/min until failure. Peak load was recorded to compute shear strength, with triplicate averages reported. Specimens were preconditioned at 25 °C for 4 h prior to testing.

#### 2.3.7. Permeability Test

Water resistance of the interlayer was evaluated using a pavement permeability tester compliant with Standard Test Methods for Asphalt and Asphalt Mixtures in Highway Engineering (JTG E20-2011). Specimens from Section 2.2 were sealed at the edges to prevent lateral leakage. The average permeability coefficient from three specimens characterized the anti-seepage performance.

#### 2.3.8. Three-Point Bending Fatigue Test

Fatigue behavior of the fiber-reinforced RAP interlayer was investigated via three-point bending tests. Specimens conditioned at 15 °C for 4 h were cyclically loaded under a stress intensity ratio of 0.4 using an MTS testing machine (MTs Systems Corporation, Eden Prairie, MN, USA) with 10 Hz sinusoidal loading. Fatigue life was determined as the number of cycles to failure, averaged across three specimens.

#### 2.3.9. DIC-Based Flexural Strength Test

**Figure 4:**
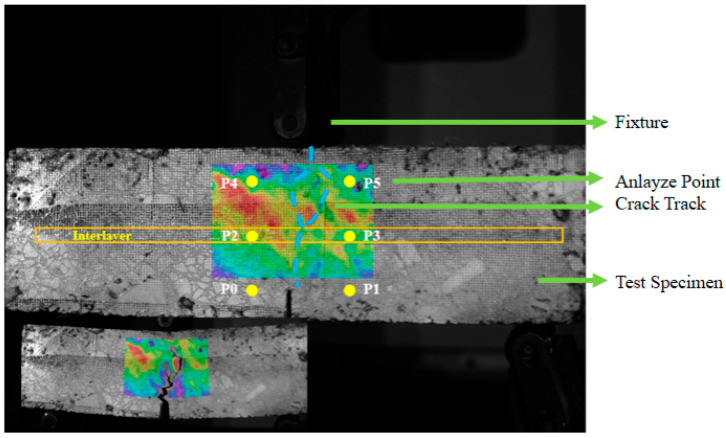
Selection of analysis points.

To elucidate crack resistance mechanisms, DIC-enhanced three-point bending tests were performed. Specimens preconditioned at 0 °C for 4 h were loaded at 50 mm/min on a SANS universal testing machine. DIC technology tracked real-time surface deformation, capturing crack propagation from a 15 mm-deep pre-notch. Six analysis points were selected at 26 mm vertical intervals from the notch tip (Figure 4), with displacement variations quantitatively analyzed to characterize crack width and propagation patterns, revealing the interlayer’s anti-cracking mechanisms.

## 3. Effect of USP on the Properties of Rubberized Asphalt

### 3.1. Three Major Indicators

#### 3.1.1. Influence of USP Warm-Mix Modifier on Penetration of Rubberized Asphalt

**Figure 5:**
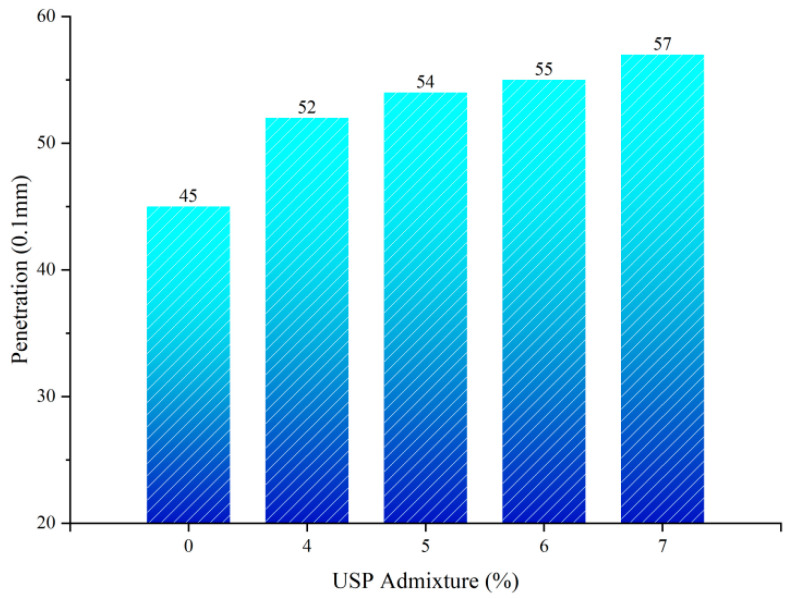
Penetration test results of USP-modified Penetration of Rubberized Asphalt.

Figure 5 presents the penetration test results. The USP warm-mix modifier exhibited significant regulatory effects on the penetration of rubberized asphalt. The baseline specimen showed a penetration value of 45 (0.1 mm), which increased monotonically with rising USP admixture content. This behavior stems from the intercalation of long-chain alkane structures in the modifier with asphalt colloids, which dilutes the asphaltene associative system and disrupts the three-dimensional network structure, thereby reducing the cohesive energy density [26]. When the USP content increased from 0% to 5%, penetration rose from 45 (0.1 mm) to 54 (0.1 mm), representing a 20.0% enhancement. Further increasing the dosage to 7% yielded only a marginal improvement to 57 (0.1 mm), with a reduced growth rate of 5.56%. This nonlinear progression suggests saturation in free volume expansion of the asphalt colloidal system beyond 5% admixture, where localized aggregation of modifier molecules likely diminishes their marginal efficacy in asphaltene dispersion.

#### 3.1.2. Influence of USP Warm-Mix Modifier on Ductility of Rubberized Asphalt

**Figure 6:**
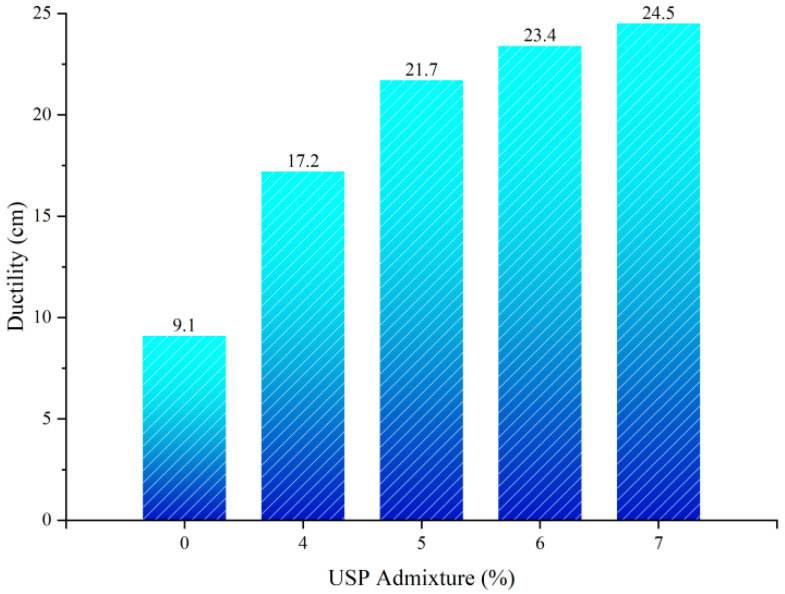
Ductility test results of USP-modified rubberized asphalt.

Figure 6 displays the ductility test results of rubberized asphalt under varying USP admixture levels. The incorporation of the warm-mix modifier markedly enhanced the low-temperature ductility of the material. Ductility values exhibited nonlinear growth with increasing USP content, reaching 21.7 cm at 5% admixture—a 141.1% increase from the baseline—and further rising to 24.5 cm at 7%, albeit with a diminished growth rate of 12.9%. This enhancement arises from the modifier’s ability to weaken intermolecular forces within resin-asphaltene aggregates and amplify chain segment mobility [27]. Beyond 5% admixture, interfacial compatibility approaches saturation, leading to attenuated ductility improvement.

#### 3.1.3. Influence of USP Warm-Mix Modifier on Softening Point of Rubberized Asphalt

**Figure 7:**
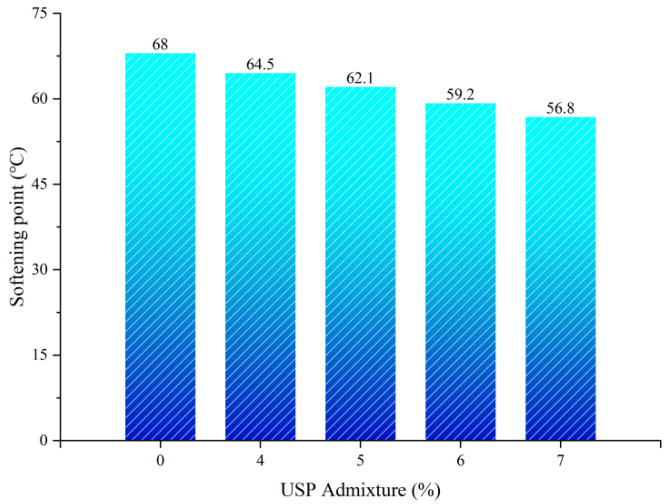
Softening point test results of USP-modified rubberized asphalt.

As shown in Figure 7, the softening point of rubberized asphalt decreased linearly with USP admixture. The baseline specimen had a softening point of 68 °C, which declined to 62.1 °C at 5% admixture (8.7% reduction) and further dropped to 56.8 °C at 7% (16.5% total reduction). This reduction is attributed to light components in the modifier diluting asphaltene colloidal clusters and weakening crosslinking density within the three-dimensional network, thereby compromising thermal stability.

### 3.2. Low Temperature Rheological Properties

**Figure 8:**
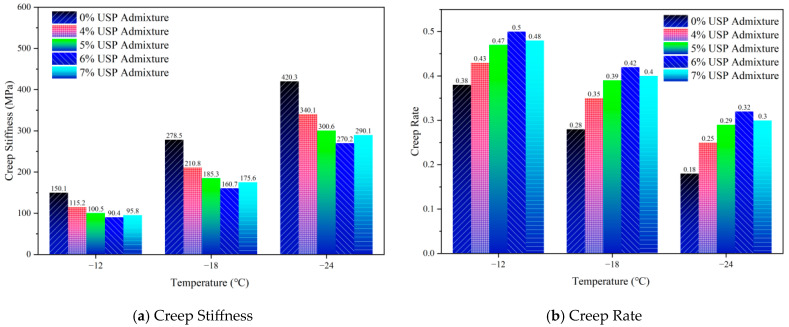
Low-temperature rheological properties of USP-modified rubberized asphalt.

To systematically evaluate the influence of USP admixture content on the low-temperature performance of rubberized asphalt, this study employed bending beam rheometer (BBR) tests to analyze variations in creep stiffness (S) and creep rate (m) across different modifier dosages and temperatures. The results, illustrated in Figure 8, demonstrate significant dose-dependent behavior. Unmodified rubberized asphalt exhibited an S-value of 420.30 MPa and an m-value of 0.18 at −24.00 °C, indicative of high rigidity and brittleness prone to thermal contraction cracking.

Increasing USP content markedly reduced S-values while elevating m-values, confirming enhanced viscoelasticity and stress relaxation capacity. At 6.00% admixture, S decreased to 270.20 MPa and m increased to 0.32, suggesting optimal dynamic equilibrium between modifier molecular chains and rubber particles via physical entanglement and hydrogen bonding. This balance effectively mitigates brittle fracture induced by glass transition while retaining viscous flow to relieve thermal stress. However, further increasing dosage to 7.00% caused S to rebound to 290.10 MPa and m to decline to 0.30, likely due to phase separation or aggregation disrupting the continuous matrix.

Notably, USP modification significantly reduced temperature sensitivity. At 6.00% dosage, the S-value increased by 198.89% as temperature dropped from −12.00 °C to −24.00 °C—substantially lower than the 280.01% increase in unmodified samples. Concurrently, m-value decreased by 36.00%, outperforming the 52.63% reduction in controls. These findings underscore USP’s efficacy in enhancing low-temperature toughness and stress dissipation, thereby reducing cracking risks.

Samples with 5.00% and 6.00% USP admixture demonstrated optimal comprehensive performance, balancing superior crack resistance with cost-effectiveness for large-scale engineering applications.

### 3.3. High Temperature Rheological Properties

**Figure 9:**
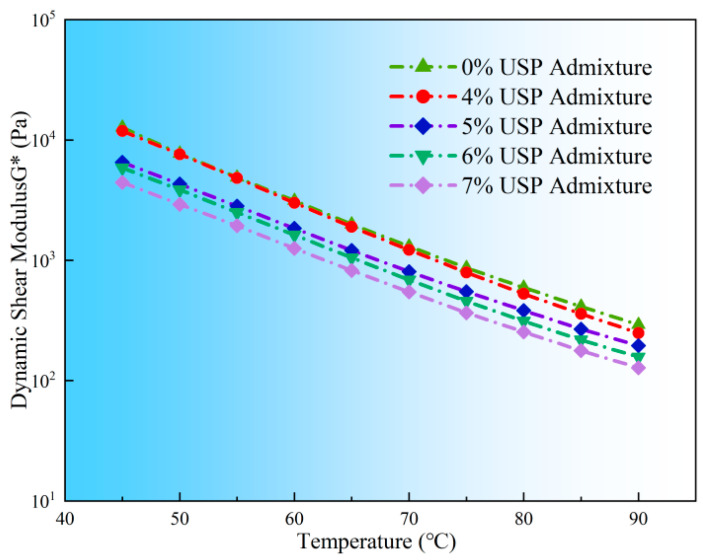
Variations in complex shear modulus of rubberized asphalt with USP admixture.

Figure 9 illustrates the temperature-dependent variations of G* for rubberized asphalt with different USP dosages. All samples exhibited significant thermal softening, characterized by a marked decline in G* with rising temperatures. The unmodified control sample showed a G* value of 14,300 Pa at 45 °C, which plummeted to 305 Pa at 90 °C—a 97.9% reduction, highlighting the profound temperature sensitivity of material stiffness. Increasing USP content further reduced G* values. At 70 °C, the 5% admixture sample demonstrated a G* of 746 Pa, representing a 46.7% decrease compared to the unmodified sample. This reduction is attributed to the modifier’s dilution effect on the three-dimensional network structure of rubber particles [28]. Notably, at 6% admixture, partial disruption of physical entanglement between rubber and base asphalt led to structural relaxation and accelerated modulus decline.

**Figure 10:**
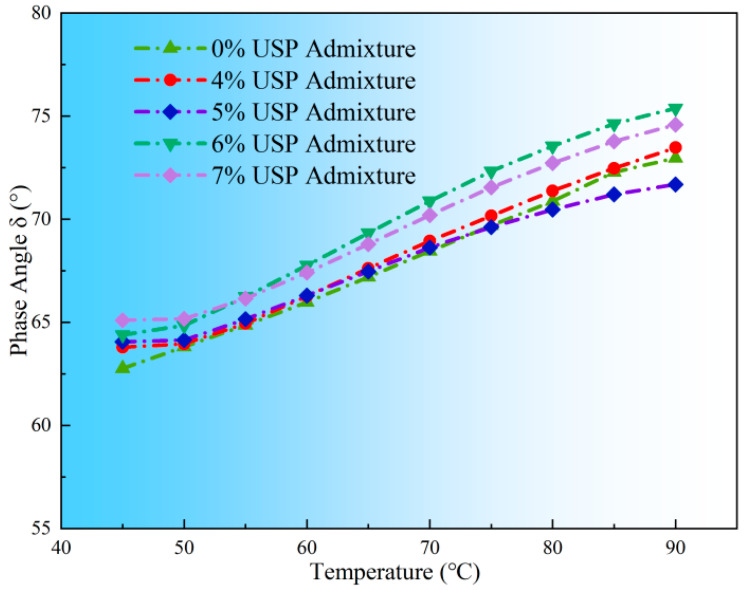
Variations in phase angle of rubberized asphalt with USP admixture.

Phase angle (δ), a critical indicator of viscoelastic balance, exhibited temperature- and dosage-dependent trends (Figure 10). All samples showed increasing δ values with temperature, reflecting a gradual shift from elastic-dominated to viscous-dominated behavior. At 70 °C, the unmodified sample had a δ of 70.30°, while the 7% admixture sample reached 72.05°, indicating enhanced viscous response. Microscopically, higher USP content weakened interfacial interactions between rubber phases and base asphalt, inducing Newtonian fluid-like rheological behavior [29]. While beneficial for workability, this shift compromises structural stability at high temperatures, increasing susceptibility to permanent deformation.

**Figure 11:**
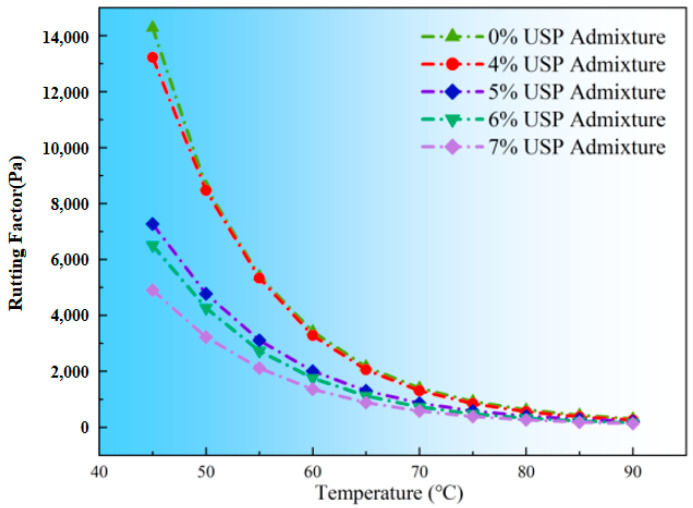
Variations in rutting factor of rubberized asphalt with USP admixture.

The rutting factor (G*/sinδ), a key metric for evaluating resistance to permanent deformation, displayed exponential decay with temperature (Figure 11). At 45 °C, the unmodified sample exhibited a G*/sinδ of 14,300 Pa, decreasing to 305 Pa at 90 °C. Performance degradation intensified at admixture levels exceeding 5%. For instance, at 70 °C, G*/sinδ values were 746 Pa (5%), 726 Pa (6%), and 581 Pa (7%), demonstrating nonlinear deterioration. This suggests that high USP dosages disrupt the continuity of rubber networks, significantly impairing rutting resistance and elevating early-stage pavement distress risks.

While USP modification enhances the workability of rubberized asphalt, excessive dosages (>5%) destabilize its viscoelastic balance and structural integrity. Optimal engineering performance requires balancing construction feasibility with high-temperature durability, recommending a maximum USP admixture of 5% to mitigate premature rutting while maintaining operational efficiency.

## 4. Effect of USP on the VOCs Emission of Rubberized Asphalt

**Figure 12:**
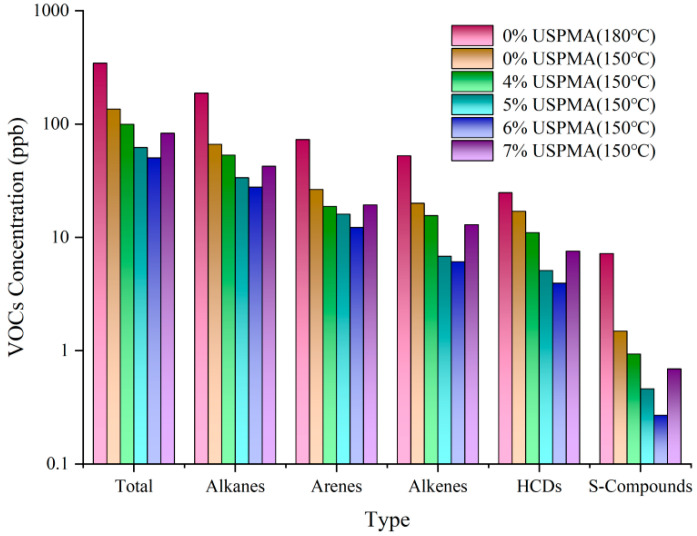
VOC concentrations of USP-modified rubberized asphalt.

Volatile organic compounds (VOCs), the primary pollutants emitted during rubberized asphalt hot-mix processes, directly influence ozone formation potential (OFP) and carcinogenic risk indices (CRI), posing significant ecological and health hazards. This study quantitatively analyzed VOC emission patterns under varying mixing temperatures and USP dosages (Table 6 and Figure 12) to elucidate the suppression mechanisms of warm-mix technology.

The experimental data in Table 6 and Figure 12 demonstrate significant regulatory effects of mixing temperature and USP admixture on VOC emissions from rubberized asphalt. Unmodified rubberized asphalt emitted a total VOC concentration of 344.72 mg/m^3^ at 180 °C, which decreased by 60.7% to 135.60 mg/m^3^ at 150 °C. This temperature reduction effectively suppressed rubber particle pyrolysis and the volatilization of light components, particularly alkenes and alkanes, with reductions of 61.92% and 64.66%, respectively.

Under the 150 °C mixing condition, VOC emissions exhibited a nonlinear trajectory with increasing USP dosage. Total VOC concentrations decreased to 99.44 mg/m^3^ (26.7% reduction) at 4% admixture and reached a minimum of 50.27 mg/m^3^ (62.9% reduction compared to the 150 °C baseline) at 6% dosage. However, a 65.0% rebound to 82.95 mg/m^3^ occurred at 7% admixture [30]. This threshold effect arises from dual mechanisms: at dosages ≤6%, USP suppresses volatilization of light components (e.g., alkanes and arenes) through physical encapsulation effects while reducing rubber particle pyrolysis via lowered asphalt viscosity.

Conversely, exceeding 6% admixture leads to saturation of the encapsulation layers and shear-induced thermodynamic instability, triggering secondary release of volatiles due to disrupted interfacial equilibrium. Sulfur-containing compounds exhibited phased reductions, declining from 1.49 mg/m^3^ (unmodified at 150 °C) to 0.27 mg/m^3^ at 6% (81.9% reduction), with a minor rebound to 0.69 mg/m^3^ at 7%, likely due to active site shielding caused by USP molecular aggregation. Despite fluctuations, sulfur emissions remained substantially lower than in unmodified systems.

The emission hierarchy was: 6% USP (150 °C) < 5% USP (150 °C) < 7% USP (150 °C) < 4% USP (150 °C) < Unmodified (150 °C) < Unmodified (180 °C). For optimal environmental and engineering performance, a mixing temperature of 150 °C with 6% USP admixture is recommended, achieving an 82% reduction in total VOCs and toxicity indices compared to the 180 °C baseline. Strict adherence to ≤6% USP dosage is critical to avoid rebound risks from side reactions.

## 5. Effects of USP on the Performance of Fiber-Reinforced Rubberized Asphalt RAP Interlayer

### 5.1. Bonding Strength

**Figure 13:**
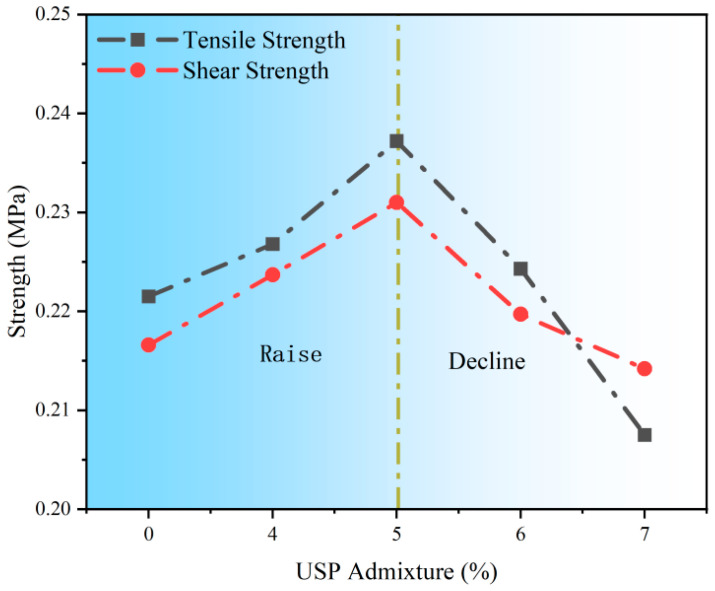
Bonding strength test results of USP-modified fiber-reinforced RAP interlayer.

The bonding strength variations with USP admixture content are illustrated in Figure 13. Based on pull-out and shear strength results, USP dosage exhibits a nonlinear regulatory effect on interfacial mechanical performance, with an optimal content identified. At 5% USP admixture, the pull-out strength peaks at 0.2372 MPa (7.09% increase from the baseline), and shear strength reaches 0.2310 MPa (6.65% improvement). This enhancement stems from the modifier’s physical plasticization effect, which reduces asphalt viscosity, promotes asphalt-aggregate interfacial wetting, and forms a dense bonding layer. However, exceeding 5% dosage degrades performance: pull-out and shear strengths decline by 12.52% and 7.27%, respectively, at 7% admixture. This deterioration arises from reduced asphalt cohesive strength due to excessive softening point depression, weakened mechanical interlocking, and dissociation of the asphaltene three-dimensional network caused by over-plasticization, ultimately lowering interfacial adhesion energy.

### 5.2. Water Permeability

**Figure 14:**
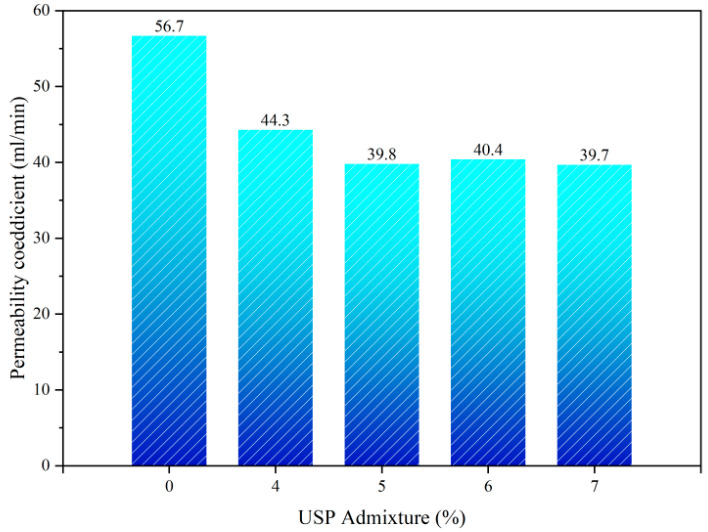
Permeability test results of USP-modified fiber-reinforced RAP interlayer.

Water permeability is a critical indicator of interlayer waterproofing. Permeability coefficient tests (Figure 14) reveal that increasing USP admixture to 5% reduces seepage rates from 56.7 mL/min to 39.8 mL/min (29.81% reduction). Beyond 5%, rates stabilize near 40 mL/min with minimal fluctuations (<1%), indicating a threshold for waterproofing optimization. The USP modifier enhances asphalt fluidity and adhesion, enabling uniform filling of aggregate voids to form a dense anti-permeability layer. Studies [31] suggest that active components in the modifier optimize molecular alignment, reducing pore connectivity and blocking water infiltration paths. However, exceeding 5% admixture induces localized flocculation in the asphalt colloidal structure due to component oversaturation, limiting further densification despite increased dosage.

### 5.3. Flexural Strength

**Figure 15:**
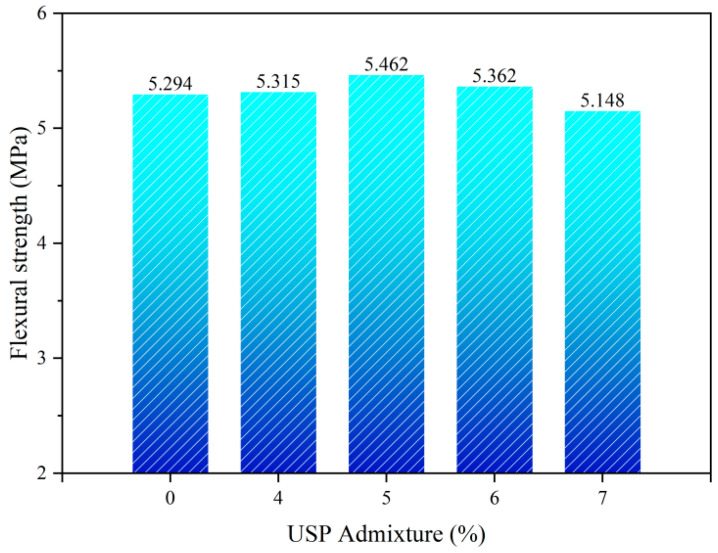
Flexural strength test results of USP-modified fiber-reinforced RAP interlayer.

Figure 15 illustrates the effects of USP admixture on the flexural strength of the fiber-reinforced RAP interlayer. Flexural strength initially increased and subsequently decreased with rising USP content. At 5% admixture, the flexural strength peaked at 5.462 MPa, representing a 3.17% improvement over the baseline. However, increasing the dosage to 7% reduced the strength to 5.148 MPa (5.75% decline). This enhancement is attributed to the modifier’s optimization of asphalt rheological properties and adhesion, facilitating uniform aggregate coating and forming a dense interfacial transition zone, thereby improving structural stiffness. Active components in the modifier enhance molecular crosslinking density, establishing a three-dimensional elastic network that delays crack propagation. Excessive admixture (>5%), however, disrupts the asphalt colloidal equilibrium, inducing phase separation [32], which compromises cohesive strength and interfacial bonding efficiency.

### 5.4. Fatigue Performance

**Figure 16:**
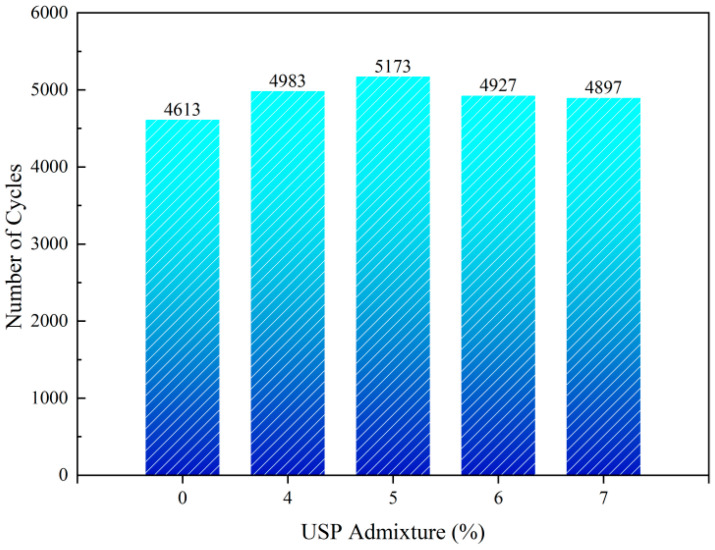
Fatigue strength test results of USP-modified fiber-reinforced RAP interlayer.

Figure 16 demonstrates the impact of USP dosage on the fatigue strength of the interlayer. Fatigue life exhibited a unimodal trend, peaking at 5173 cycles (12.14% increase) with 5% USP admixture, followed by a 5.34% reduction to 4897 cycles at 7%. Optimal USP content (5%) enhances ductility and penetration, improving material flexibility and stress relaxation capacity, which effectively redistributes load-induced stresses and retards crack growth [33]. Excessive admixture lowers the softening point, diminishing high-temperature deformation resistance, while surplus low-molecular-weight components destabilize the colloidal structure, accelerating plastic deformation accumulation under cyclic loading and hastening fatigue failure [17]. These findings confirm that 5% USP admixture optimally balances performance, providing a viable strategy to enhance the durability of RAP-based Interlayer.

### 5.5. Fracture Mechanism Analysis Based on DIC Technology

**Figure 17:**
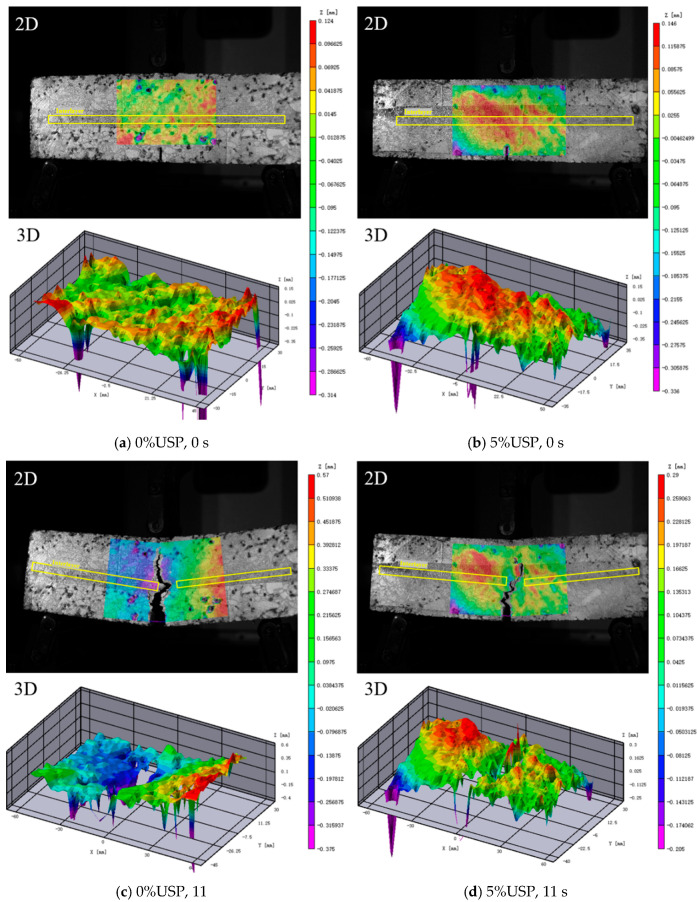
Failure images of specimens under VIC system at different time points.

To visually assess the influence of USP admixture on the flexural performance of the RAP interlayer, a three-dimensional digital image correlation (DIC) system was employed to analyze deformation patterns during crack propagation. Figure 17 compares bending process images of RAP interlayer specimens with 0% and 5% USP admixture at different time points.

At 11 s, the 5% USP-modified specimen exhibited a crack length of 6.18 cm, significantly shorter than the 6.53 cm in the unmodified specimen, while the crack width at the specimen base decreased from 1.67 cm to 0.92 cm. These results confirm that USP modification enhances crack resistance by improving asphalt-aggregate interfacial bonding and optimizing the mechanical properties of the interfacial transition zone, thereby strengthening overall structural integrity.

**Figure 18:**
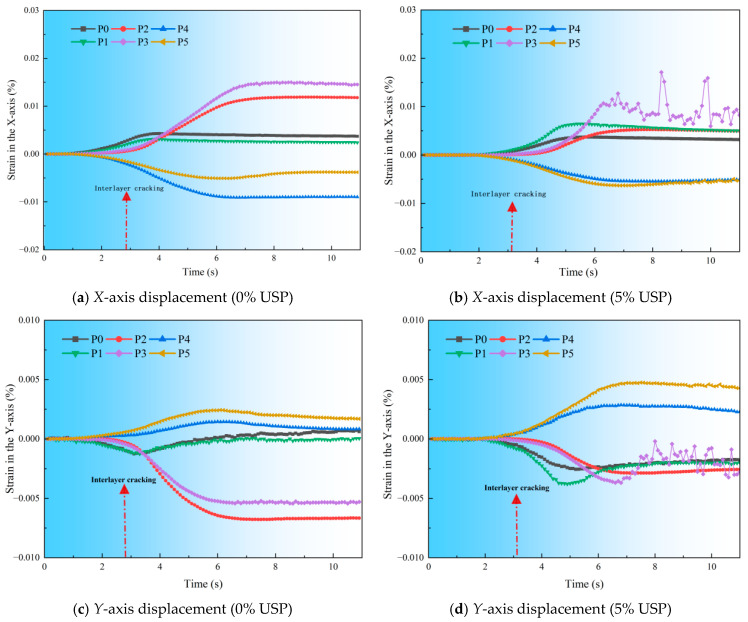
Relative displacements for RAP interlayer with varying USP admixture.

To further investigate crack propagation dynamics, relative displacement changes along the X- and Y-axes at selected measurement points (Figure 17) were quantified and plotted in Figure 18.

During the 5–11 s propagation phase, *X*-axis displacements at Measurement Points 1 and 4 decreased by 56.6% (0.4749% vs. 1.0954%) and 35.9% (0.8694% vs. 1.3558%), respectively. *Y*-axis displacements reduced by 59.5% (0.262% vs. 0.656%) and 57.8% (0.222% vs. 0.526%) at the same points. At the upper interlayer (Measurement Point 2), *X*-axis displacement decreased by 41.9% (0.515% vs. 0.886%), while Measurement Point 5 exhibited a 33.3% reverse increase (0.576% vs. 0.432%), revealing anisotropic constraint effects induced by the modifier.

Collectively, USP-modified specimens demonstrated consistently smaller displacements across both axes compared to unmodified counterparts, particularly at the interlayer and upper-left regions. This confirms that the USP-modified interlayer more effectively inhibits crack propagation, mitigates upward crack transmission, and enhances global crack resistance.

## 6. Conclusions

This study systematically investigated the effects of the USP warm-mix modifier on rubberized asphalt and fiber-reinforced RAP interlayers, elucidating its role in enhancing material performance, crack resistance, and emission control. The key findings are as follows:

1. USP significantly improves the low-temperature flexibility and workability of rubberized asphalt by reducing viscosity and enhancing flow, with an optimal dosage of 5% balancing low-temperature performance and high-temperature stability.
2. USP effectively suppresses VOC emissions during high-temperature construction. At 150 °C, a 6% dosage reduced total VOC emissions by 62.9% compared to unmodified asphalt at the same temperature and by 85.4% relative to 180 °C unmodified asphalt, attributed to its surface-active properties and superior dispersion at lower temperatures.
3. For the RAP fiber-reinforced interlayer, a 5% USP dosage maximized key performance metrics: pull-out strength (+7.09%), shear strength (+6.65%), moisture damage resistance (+29.81%), flexural strength (+3.17%), and fatigue life (+12.14%), reflecting USP’s enhancement of asphalt-aggregate adhesion and interfacial cohesion.
4. DIC-based analysis demonstrated that USP mitigates crack initiation and propagation by optimizing asphalt rheology and interfacial bonding, improving stress distribution at crack tips, and significantly enhancing crack resistance, thereby extending pavement service life through structural reinforcement.

Novelty and limitations: This study is the first to systematically integrate USP warm-mix modification with rubberized asphalt RAP interlayers, quantifying threshold effects on low-/high-temperature performance, VOC emission reduction, and interface reinforcement. However, the long-term durability and environmental adaptability under field conditions, particularly with varying RAP aging levels and climate scenarios, require further investigation.

## Figures and Tables

**Table 1:** Key properties of rubber asphalt.

Type	Detection Items		Test Results	Methods (JTG E20-2011 [22])
Rubber asphalt	Penetration 25 °C, 100 g, 5 s (0.1 mm)		45	T 0604
	Penetration index PI		0.8	-
	Ductility 5 cm/min, 5 °C (cm)		90	T 0605
	Softening point (°C)		68	T 0606
	Elastic recovery 25 °C (%)		86	T 0662
	Storage stability segregation (°C)		1.8	T 0655
	Residue after RTFOT (163 °C, 85 min)	Mass loss (%)	−0.06	T 0610
		Penetration ration 25 °C (%)	76	
		Residual ductility 15 °C (cm)	28	
Asphalt in RAP	Penetration at 25 °C (100 g, 5 s, 0.1 mm)		17	T 0604
	Ductility at 5 °C (cm)		Brittle	T 0605
	Ductility at 15 °C (cm)		11	T 0605
	Softening Point (°C)		71	T 0606

**Table 2:** Properties of basalt crushed stone.

Detection Items	Results	Standard	Methods (JTG 3432-2024 [23])
Los Angeles abrasion loss (%)	26	≤35	T 0317
Crushing value (%)	16	≤30	T 0316
Needle-like content (%)	11	≤20	T 0312
Asphalt Adhesion (grade)	6	>4	T 0323
Moisture content (%)	1.4	≤2	T 0304
PSV	18	≤38	T 0321
Durability (%)	8	≤12	T 0314

**Table 3:** Physical properties of basalt fibres.

Length (mm)	Single Fiber Diameter (μm)	Density (kg/m^3^)	Fracture Elongation Rate (%)	Elastic Modulus (GPa)	Pull-Out Strength (MPa)	Moisture Content (%)
20	15	2645	2.4–3.2	90–110	3200–4600	0.06

**Table 4:** Technical specifications of USP warm-mix modifier.

Item	Unit	Specification	Methods
Appearance	/	Black or purple-black paste	Visual inspection
Odor	/	Odorless	Olfactory test
Flash point (open cup)	°C	>120	JTG E20 T0611
Density	g/cm^3^	0.94–0.99	JTG E20 T0603
Moisture content	%	<0.2	GB/T 260-2016 [24]
Ash content	%	<0.8	JTG E20 T0614

**Table 5:** Mix design of USP Warm-Mix Modified Rubberized Asphalt.

Sample ID	Base Asphalt (%)	Crumb Rubber (%)	USP Warm-Mix Additive (%)
RAU-0	90	10	0
RAU-4	90	10	4
RAU-5	90	10	5
RAU-6	90	10	6
RAU-7	90	10	7

**Table 6:** VOC concentrations of rubberized asphalt under different conditions (ppb).

USP Admixture	0%	0%	4%	5%	6%	7%
Temperature	180 °C	150 °C	150 °C	150 °C	150 °C	150 °C
Alkanes	187.42	66.23	53.21	33.63	27.71	42.40
Arenes	72.84	26.45	18.73	16.00	12.24	19.34
Alkenes	52.47	19.98	15.55	6.82	6.10	12.94
HCD *	24.79	16.99	11.01	5.11	3.94	7.57
S-Compounds *	7.20	1.49	0.94	0.46	0.27	0.69
Total	344.72	135.60	99.44	62.02	50.27	82.95

E.g., * sulfur-containing compounds (S-Compounds); * Hydrocarbon Derivatives (HCDs).

## Data Availability

The original contributions presented in this study are included in the article. Further inquiries can be directed to the corresponding author.
